# Supplementary material for: MetaRibo-Seq measures translation in microbiomes
Source: Nat Commun. 2020 Jun 29;11:3268. doi: 10.1038/s41467-020-17081-z (PMC7324362; doi:10.1038/s41467-020-17081-z)
Supplement: Supplementary file 10 — Supplementary Data 7 [file 41467_2020_17081_MOESM10_ESM.zip › File2/Confidence_High_Taxonomy/30537_out.krona.html]

Javascript must be enabled to view this page.

members
magnitude
magnitudeUnassigned
count
unassigned
taxon
rank

30537\_out

71

71
2
superkingdom

71
1239
phylum

class
186801
71

71
order
186802

1

SRS012849\_contig\_number\_2391
1897045
species

70
family
541000

1
genus
292632


SRS018656\_contig\_number\_11660
1
species
214851

69
genus
1263

2293179
species

SRS014287\_contig\_number\_18798SRS014923\_contig\_number\_contig-100\_9.246486SRS015190\_contig\_number\_16947SRS015578\_contig\_number\_16670SRS018427\_contig\_number\_20502SRS018836\_contig\_number\_27090SRS019030\_contig\_number\_19061SRS043411\_contig\_number\_18071SRS050925\_contig\_number\_34861SRS054956\_contig\_number\_16532SRS062701\_contig\_number\_1941SRS064757\_contig\_number\_contig-100\_5792.93057SRS077335\_contig\_number\_5320SRS077502\_contig\_number\_contig-100\_1.117637SRS098644\_contig\_number\_49722SRS098655\_contig\_number\_12204SRS104084\_contig\_number\_871SRS147557\_contig\_number\_32776SRS147919\_contig\_number\_contig-100\_722.121748SRS148196\_contig\_number\_41195
20

species
40518

SRS011302\_contig\_number\_contig-100\_17.167315SRS016203\_contig\_number\_916SRS024132\_contig\_number\_48732SRS053335\_contig\_number\_18047SRS077641\_contig\_number\_contig-100\_57.125865SRS098073\_contig\_number\_1389SRS104400\_contig\_number\_575SRS140492\_contig\_number\_32930SRS893369\_contig\_number\_4213
9

species
2293209
5

SRS013476\_contig\_number\_29798SRS016132\_contig\_number\_contig-100\_58.45687SRS065504\_contig\_number\_contig-100\_7.226226SRS097889\_contig\_number\_40079SRS143780\_contig\_number\_32285

6

SRS018656\_contig\_number\_11640SRS058145\_contig\_number\_contig-100\_24036.24037SRS078242\_contig\_number\_7650SRS078419\_contig\_number\_10216SRS104975\_contig\_number\_11180SRS147346\_contig\_number\_68371
41978
species


SRS011239\_contig\_number\_26911SRS012969\_contig\_number\_36706SRS013687\_contig\_number\_31309SRS016495\_contig\_number\_20081SRS016954\_contig\_number\_27104SRS017307\_contig\_number\_9248SRS018623\_contig\_number\_14509SRS018936\_contig\_number\_11181SRS023526\_contig\_number\_37347SRS023914\_contig\_number\_contig-100\_796.161142SRS048981\_contig\_number\_2005SRS049773\_contig\_number\_37913SRS050998\_contig\_number\_627SRS053214\_contig\_number\_13268SRS053335\_contig\_number\_contig-100\_987.109561SRS063985\_contig\_number\_10087SRS075021\_contig\_number\_4247SRS077194\_contig\_number\_contig-100\_19.192039SRS077730\_contig\_number\_8029SRS1041036\_contig\_number\_1127SRS1041145\_contig\_number\_contig-100\_9216.118920SRS1055038\_contig\_number\_contig-100\_13016.13017SRS146813\_contig\_number\_contig-100\_449.162918SRS147271\_contig\_number\_9127SRS148159\_contig\_number\_30753SRS148721\_contig\_number\_53735SRS148874\_contig\_number\_16927SRS893279\_contig\_number\_16707SRS971275\_contig\_number\_contig-100\_3875.211136
29
2293148
species
